# Nutritional and health benefits of wild and cultivated yam (*Dioscorea* spp.) species consumed in eastern Democratic Republic of Congo

**DOI:** 10.1038/s41598-025-26046-5

**Published:** 2025-11-26

**Authors:** Jean M. Mondo, Alphonse Z. Balezi, Jackson Ishara, Jean-Claude I. Mwanga Mwanga, Esther R. Matendo, Denis-Gilbert N. Bagaya, Géant B. Chuma, Paterne A. Agre, Patrick Adebola, Asrat Asfaw

**Affiliations:** 1 Department of Crop Production, Université Evangélique en Afrique (UEA), Bukavu, 3323 Democratic Republic of Congo; 2 Department of Agronomy, Université Officielle de Bukavu (UOB), Bukavu, 570 Democratic Republic of Congo; 3 Department of Botany, Université Officielle de Bukavu (UOB), Bukavu, 570 Democratic Republic of Congo; 4 Department of Food Science and Technology, Université Evangélique en Afrique (UEA), Bukavu, 3323 Democratic Republic of Congo; 5 Laboratory of Plant Systematics and Taxonomy, Centre de Recherche en Sciences Naturelles de Lwiro, Lwiro, Democratic Republic of Congo; 6 International Institute of Tropical Agriculture (IITA), Ibadan, 5320 Nigeria; 7 International Institute of Tropical Agriculture (IITA), Abuja, 901101 Nigeria

**Keywords:** Antioxidants, *Dioscorea* species, Ethno-botanical study, Nutritional quality, Phytonutrients, Biochemistry, Ecology, Genetics, Plant sciences, Health care

## Abstract

Yam (*Dioscorea* spp.) is a tuber-producing crop and an important staple in the tropics and subtropics, valued for its nutritional, health and sociocultural relevance. However, there is limited awareness of its nutritional and health benefits among local communities in eastern Democratic Republic of Congo (DRC), prompting its underutilization. This study aimed to (a) document the diversity of cultivated and semi-domesticated yam species in eastern DRC; (b) explore indigenous knowledge on their nutritional and health benefits; and (c) assess the nutrient composition and antioxidant activity of major yam species consumed in the region. Data collection involved ethno-botanical surveys, plant specimen collections, interviews with 123 community members, and physicochemical profiling using standard analytical methods. Ten yam species were documented in eastern DRC, including two domesticated species, with distribution patterns shaped by biophysical conditions and cultural factors. Species richness was higher in highland forest zones compared to lowland savannas. Local communities use yam to treat more than 15 health conditions, including stomach pains, food intoxications, skin wounds and infections, reproductive abnormalities, immune system deficiency, respiratory challenges, diabetics, etc. Physicochemical composition analysis revealed interspecific variations potentially contributing to their nutritional and therapeutic relevance by local communities. Notably, *Dioscorea dumetorum* tubers exhibited the highest protein content (8.7 g/100 g) and potassium concentration (240.2 mg/100 g), *D. praehensilis* was rich in carbohydrates (80.9 g/100 g), and *D. bulbifera* had highest calcium concentration (40.1 mg/100 g), underscoring the yam nutritional significance in the local diet. All yam species encompassed saponins, terpenoids, and alkaloids, though *D. praehensilis* and purple *D. alata* had the highest antioxidant activity that exceeded 90% inhibition level. These findings provide valuable insights on yam diversity and their potential to sustain nutritious healthy diets in eastern DRC.

**Supplementary Information:**

The online version contains supplementary material available at 10.1038/s41598-025-26046-5.

## Introduction

Yam (*Dioscorea* spp.) is a generic name referring to approximately 600 species of the *Dioscorea* genus (Plum. ex L.), cultivated for its starchy underground or aerial tubers in tropical and subtropical regions of Africa, Asia, and America^1^. Among these species, only 11 are cultivated for food and income, while others remain in wild undomesticated but often used by hunter-gathers to mitigate food shortages during lean periods and for traditional rituals and medicine^2–5^. Beyond its role as a staple food, substantial evidence highlights yam’s nutritional and therapeutic contributions to human health. It serves as an important source of dietary energy, dietary fiber, protein, vitamins (C and B), essential minerals (potassium, manganese, calcium, etc.), while maintaining a low fat content^6–8^. These nutrients support key physiological functions, including energy metabolism, nervous system health, muscle function, blood pressure, blood sugar level regulation, bone health, intestinal transit regulation, diabetics, and satiety^7,9,10^. Besides, yam is deeply embedded in cultural and spiritual traditions of societies who depends on it, often considered as a sacred crop symbolizing fertility, prosperity, and connection to the land^2^. Yam also contains bioactive phytochemicals with antioxidant activity, including polyphenols, sterols, diosgenin, flavonoids, saponins, alkaloids, and terpenoids. These compounds exhibit free radical scavenging activity, reducing oxidative stress and contributing to the prevention and treatment of a range of health conditions in traditional and modern medicines, depending on their specific chemical nature, concentration, and bioavailability^10–14^.

The Democratic Republic of Congo (DRC) is a biodiversity hotspot^15^, home to over 10,000 plant species, including 23 *Dioscorea* species from different sections^16^. Several *Dioscorea* species serve as important food crops across various regions of DRC, cultivated for their starchy underground tubers and aerial bulbils^17–19^. However, research on *Dioscorea* species remains limited in eastern DRC, particularly in South-Kivu province, resulting in a poor valuation and understanding of yam species diversity in the region. This knowledge gap has led to species misidentifications among both farmers and the local scientific community, with different *Dioscorea* species and varieties often sharing the same vernacular names^19^. There is, therefore, an urgent need for comprehensive yam characterization to support ongoing genetic improvement efforts. Additionally, low awareness of the nutritional and health benefits of yam among the local urban and rural consumers poses a significant challenge to its wider production and utilization in eastern DRC. A World Food Program (WFP) initiative promoting indigenous foods in Southern Africa Development Community (SADC) countries highlighted the importance of nutritional education in increasing the awareness of such foods in local diet systems. However, for such efforts to be effective, messaging must be grounded on sound scientific knowledge, while being accessible and culturally relevant capable of stimulating positive behaviours at the local level^20^. Information on yam species richness, their nutritional, phytochemical, and antioxidant compositions would be necessary for biodiversity conservation efforts, plant breeding, awareness campaigns vis-à-vis yam nutritional and health benefits, and for processing and value addition initiatives.

This study, therefore, aimed at raising awareness on the nutritional and therapeutic benefits of major cultivated and wild yam species in eastern DRC. It specifically sought to (a) inventory cultivated and semi-domesticated yam species in South-Kivu; (b) document local knowledge on their nutritional and health benefits; and (c) assess nutrient composition and antioxidant activity of major local yam species.

## Materials and methods

### Study site

**Fig. 1:**
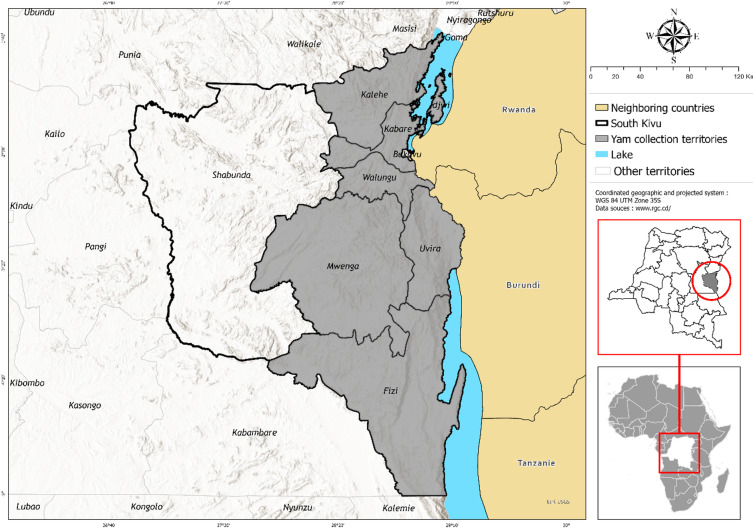
Study area, South-Kivu, Eastern DRC.

Ethno-botanical surveys were conducted in Kabare and Kalehe territories in the highlands of the South-Kivu province, eastern DRC (Table 1). These are territories neighboring the Kahuzi Biega National Park where autochthonous pygmies are located, an ethnic group depending on the forest for food, income, and medication^21^. In addition to autochthonous pygmies, other ethnic groups in the surveyed areas include Bashi, Bahavu, and Batembo all having yam as a cultural and social symbol. Administrative zones directly involved in the ethno-botanical study are Irhambi-Katana, Bugorhe, and Miti (Kabare) and Bitale, Kalima, Buloho, and Kalonge (Kalehe). Villages harboring autochthonous pygmies, among those surveyed, are Buyungule (Miti) and Cahoboka (Irhambi-Katana) in Kabare and Hembe (Bitale) in Kalehe territory. Regardless of the ethnic group, agriculture is the main economic activity in these areas, dominated by cassava, banana, common bean, sweet potato, and yam as staple crops while tea, coffee, and cinchona dominate industrial perennial crops^19^. Plant specimen collections were extended to other South-Kivu territories such as Walungu, Idjwi, Mwenga, Uvira, and Fizi (Fig. 1) to gain insights on yam diversity and spatial distribution across the province. For further details on these territories’ biophysical characteristics, see Mondo et al.^19,22^.

**Table 1:** Description of territories covered by ethnobotanical surveys.

Parameters	Kabare	Kalehe
Geographical coordinates	Long : 28°45’–28°55’ E Lat : 2°30’–2°50’ S Alt : 1460–3000 masl	Long : 28°54’–28°91’E Lat : 2.070–2.75° S Alt : 1300–3308 masl
Area (km^2^)	1960	5707
Climate	Tropical mountainous humid climate. The dry season extends from June to August, while the rainy season runs from September to May.	Mountainous to savannah tropical climate with alternating rainy (9 months) and dry (3 months) seasons.
Rainfall (mm year^− 1^)	1300–1800	1300–1800
Temperature (°C)	19.7	18–22
Soils	Dominated by Ferralsols and Cambisols. Acrisols and Nitisols can still be found in some places.	Dominance of Cambisols and Ferralsols along the Mitumba range. Presence of few Acrisols and Nitisols
Population density (habitants km^− 2^)	~ 347	*>* 300
Dominant ethnic groups	Bashi, Bahavu, and Batwa	Bashi, Bahavu, Batwa, and Batembo
Vegetation	Transitional forest with an altitude varying between 1400 and 2600 masl. The equatorial rainforest extends from ~ 850 to 1400 masl; the bamboo and *Podocarpus* forest are between 2600 and 3200 masl, while the alpine stages are between 3,200 and 3,308 masl	Grassy savannah is dominated by *Hyparrhenia* spp. and wooded savannah by *Michelsonia* spp. and *Brachystegua* spp. These vegetation types dominate at ~ 900 to 1700 masl. Above ~ 1700 masl, mountain forest appears, characterized by *Parinari excelsa* Sabine, *Symphonia globulifera* L.f., *Carapa grandiflora* Sprague, and *Macaranga* spp. alternating with tree ferns (*Cyathea manniana* Hook.) in valleys and wetlands. Mountain-top clearings can also be observed. Above ~ 1900 masl, bamboo forest (*Arundinaria alpina* K.Schum.) appears in the mountain forest, then towards the crest expanses completely dominated by bamboo.
Topography	Kabare is part of the Mitumba mountain range, with Batwa pygmies’ villages to the west and Mont Kahuzi to the northeast.	Kalehe is part of the Mitumba mountain range, rising from low altitude (~ 800 m) in the west to 3034 m (Mont Kahuzi) towards the northeast.

Long = longitude, Lat = latitude, Alt = altitude, masl = meters above sea level; Mondo et al.^21,23^; Mugumaarhahama et al.^24^.

Yam tubers used for physicochemical analyses were collected from the Université Evangélique en Afrique (UEA) Experimental Site located at Kashusha (28°47’ 45.9” E, 02°19’0.02” S, at 1717 m above sea level), in Kabare territory, eastern DRC. This experimental site experiences a bimodal tropical mountain climate (*Aw3*) with a total rainfall amount of ~ 1500 mm and an average temperature of 19.2 °C for the 2023–2024 cropping season. The site soils are of ferralitic, humus-bearing and clayey nature, underlain by basaltic rocks. Further details on Kashusha soil characteristics are provided in Table **S1**. The soil is slightly acidic (5.6), which is correlated with a high organic matter content. The carbon content and the essential bases for plant growth are all within the acceptable range for yam cultivation. These bases are available in the soil solution due to a slightly higher cation exchange capacity compared to typical tropical soils. The soil texture is clay loam according to the United States Department of Agriculture (USDA)’s textural triangle classification. Such soil texture is characterized by good water retention, ensuring adequate plant hydration. It also tends to retain nutrients better than other soil types, such as sandy soils, while maintaining ease of cultivation. The total nitrogen content remains slightly low. The calculation of the base saturation percentage (TSB) also indicates that the soil is moderately to fairly fertile (Table **S1**).

### Methods

#### Inventory of yam species in eastern DRC

Plant materials were collected under the Nagoya Protocol on Access and Benefit-sharing and were only used for research purposes. To inventory yam species across surveyed villages of eastern DRC, a 30-day field mission was conducted using two methods. Firstly, the taxonomic collection method, which involved visiting villages using a transect walk as defined by Dery et al.^25^, following horizontal axes. Plant specimen (made of tubers or plantlets) of wild and cultivated yams were collected for *ex-situ* characterization at the Lwiro Natural Science Research Centre (CRSN Lwiro), in Kabare territory. Herbarium samples were also collected using secateurs and transported to the laboratory in presses for drying, identification, and confirmation at the CRSN Lwiro herbarium. Formal identification of the plant material used in this study was undertaken by qualified personnel from the CRSN Lwiro herbarium (Mr. J.C. Ithe Mwanga-Mwanga) where voucher specimen of this material has been deposited in a publicly available herbarium. Provisional voucher/sample identifiers under which these specimens are deposited at the CRSN Lwiro Herbarium are as follows: LW_UEA5 for *Dioscorea dumetorum* (Kunth) Pax, LW_UEA2 for *Dioscorea bulbifera* L., LW_UEA1G for *Dioscorea praehensilis* Benth., LW_UEA1/MMP1 for *Dioscorea minutiflora* Engl., LW_UEA35 for *Dioscorea quartiniana* A.Rich., LW_UEA22 for *Dioscorea schimperiana* Hochst. ex Kunth, LW_UEA24 for *Dioscorea semperflorens* Uline, and LW_UEA75/24 for *Dioscorea alata* L. Updated scientific names and authors were verified for all species at https://powo.science.kew.org/. To these plant specimens were added those previously collected across all other South-Kivu territories^19^ that were being maintained at the UEA Experimental Site located at Kashusha.

#### Assessment of local knowledge on nutritional and health benefits of yams

Ethnobotanical surveys were conducted from October 2023 to January 2024, involving visiting and interviewing local ethnic communities about their knowledge on yam diversity (in terms of species), as well as its food, medicinal, or cultural importance. Formal and informal interviews were conducted with 123 community members with varying ethnic backgrounds. Respondents were sampled using a chain-reference sampling technique, also known as snowball sampling method^26^ while sample size was defined using a probabilistic saturation sampling method^27^. The study protocol was approved by the UEA Interdisciplinary Centre of Ethical Research (CIRE), Ref: CNES 039/DPSK/322PP/2023. We obtained verbal informed consent from all resource-persons and community members before interviews after ensuring the confidentiality in use of data collected and explaining the study objectives, as approved and directed by the above Institutional Review Board. Whenever possible, surveys were coupled with germplasm collection (including plantlets or tuber fragments) for *ex-situ* characterization. Collected planting materials were enveloped in paper bags with small air holes.

#### Assessment of phytochemical compositions of major yam species

*Plant material.* Laboratory assays involved five cultivars from four yam species that are local to the South-Kivu province. Of these four yam species, two were semi-domesticated, namely *D. praehensilis* Benth., locally known by its generic name as *Birongo* (represented by the Kanyaburhole variety) and *D. bulbifera* L. (represented by the wild Mange variety, for which underground and aerial tubers were collected). The two cultivated yam species included *D. alata* L. (represented by white and purple Kitende varieties) and *D. dumetorum* (Kunth) Pax, known locally as *Maliga*. Further details on used plant materials are provided in Table 2. These plant materials were sampled from the yam germplasm maintained at the UEA experimental site with a backup at the CRSN Lwiro. Planting at the Kashusha site was done in November 2023. Recommended field management was followed, including ridging, individual plant staking, fertilizer application, supplemental irrigation, regular weeding, etc. while sample materials (tubers) used for analyses were harvested in late May 2024.

**Table 2:** Yam cultivars involved in physicochemical analyses.

Vernacular name	Dialect	Species	Flesh color	Territory	Collection village	Domestication status
Maliga	Kifulero	*D. dumetorum*	Yellow	Uvira	Butole	Domesticated
Kitende	Kibembe	*D. alata*	White	Fizi	Sebele	Domesticated
Mange (Mayange)	Kihavu	*D. bulbifera*	Yellow	Idjwi	Mugote	Semi-domesticated
Kitende	Kifulero	*D. alata*	Purple	Uvira	Kahuzi	Domesticated
Kanyaburhole	Mashi	*D. praehensilis*	White	Kabare	Buyungule	Semi-domesticated

*Sample preparation.* All collected tubers were weighed, peeled, cut into slices, and then dried in an electric dryer. Dry slices were ground using a blender. The powder obtained was stored in hermetically sealed jars (tightly closed plastic boxes) at room temperature and in a dry place. This powder was thereafter shipped to Jomo Kenyatta University of Agriculture and Technology (JKUAT)’s Food Technology laboratory, in Kenya, for physicochemical analyses.

*Physicochemical analyses.* Proximate analyses and energetic values (moisture, protein, fat, crude fiber, total available carbohydrates, and ash) were determined according to the methods by the Association of Official Analytical Chemists^28^. Mineral contents (zinc, potassium, magnesium, iron, and calcium) were determined by dry ashing and atomic absorption spectrophotometer (AAS) (Shimadzu AA-7000), according to AOAC^29^ and Osborne and Voogt^30^. The β-carotene content was determined from approximately 2 g of fresh tubers following analytical methods suggested by Barba et al.^31^. Qualitative phytochemical analyses included tests for the presence of alkaloids, terpenoids (Salkowski test), and saponins. Among quantitative phytochemicals, condensed tannins were assayed according to the vanillin-hydrochloric acid method^32,33^ while flavonoids were determined using the method of Harborne and Williams^34^ and the aluminum chloride colorimetric method^35^ for qualitative and quantitative analyses, respectively. On the other hand, the total polyphenol was determined using the method of Waterman and Mole^36^, with slight modification. The radical scavenging activities of the plant extracts against 2, 2-Diphenyl-1-picryl hydrazyl (DPPH) radical (Sigma-Aldrich) were determined by UV spectrophotometer at 517 nm^37^. The following concentrations of the extracts were prepared, 0.01, 0.1, 1.0, 2.0 and 5 mg/mL in methanol (Analar grade). Vitamins C were used as the antioxidant standard at concentrations of same as the extract concentrations. More detailed methodology on physicochemical analyses is provided as **Supplementary File 1**.

### Data analyses

Descriptive statistics were performed for data collected from the ethnobotanical surveys; qualitative and quantitative data were summarized as frequencies and means ± standard deviations, respectively. ArcGIS 10.7 Esri-TM software was used to map yam species’ spatial distribution across surveyed areas of the South-Kivu province. Floristic similarity and diversity indices (Shannon-Weiner (H’), Pielou’s evenness or equitability (J), and Dominance (D)) across surveyed territories were computed using Paleontological statistics software (PAST^38^. Alternative to Student’s t test, the Wilcoxon non-parametric test was applied to assess differences in diversity between Kabare and Kalehe, two locations covered by the taxonomic collection method. The analysis of variance (ANOVA) test was applied on physicochemical data to determine significant differences among yam species for all quantitative variables. Means of physicochemical data were separated using post-ANOVA Fisher’s Least Significant Difference (LSD) test at 5% *p*-value significance level, using R 4.1.2^39^.

## Results and discussion

### Domesticated and semi-domesticated yam species in eastern DRC

**Fig. 2:**
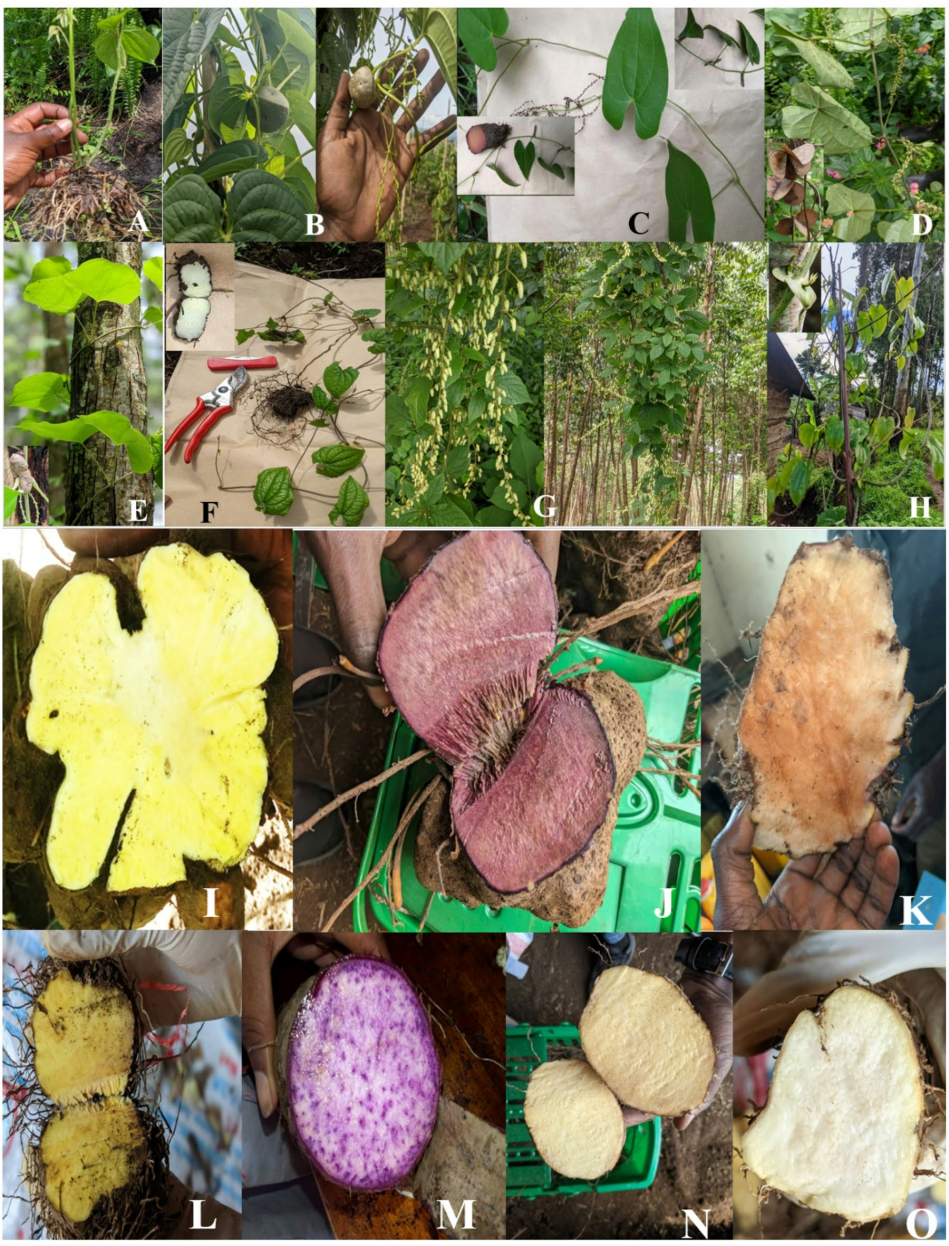
Morphological differences among some yam species inventoried in South-Kivu: (**A**) *D. dumetorum*, (**B**) *D. bulbifera*, (**C**) *D. paleata*, (**D**) *D. semperflorens*, (**E**) *D. schimperiana*, (**F**) *D. minutiflora*, (**G**) *D. quartiniana*, (**H**) *D. praehensilis*, (**I**) *D. dumetorum*, (**J**) Pink and (**K**) white *D. praehensilis*, (**L**) *D. bulbifera* underground tuber, (**M**), (**N**), (**O**) purple, yellow, and white *D. alata*, respectively.

**Fig. 3:**
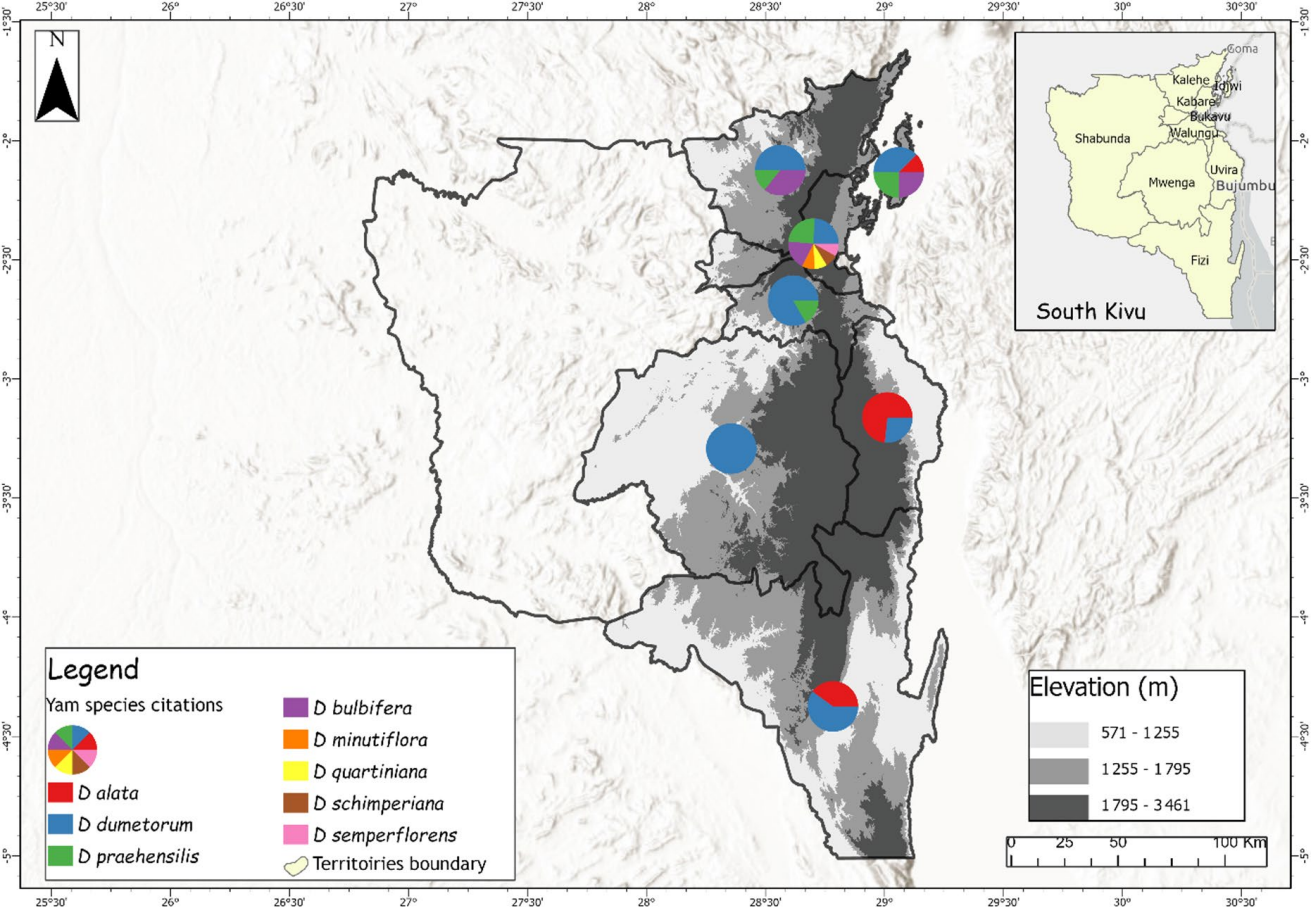
Spatial distribution of major yam species found in South-Kivu, eastern DRC.

Through extensive surveys and field observations across the study area, we collected 515 morphotypes representing 10 different *Dioscorea* species, of which *Dioscorea dumetorum* (Kunth) Pax (39.6%), *D. praehensilis* Benth. (18.8%), *D. bulbifera* L. (18.2%), and *D. quartiniana* A.Rich. (8.7%) were the most predominant. Other yam species identified in South-Kivu are *D. alata* L., *D. minutiflora* Engl., *D. schimperiana* Hochst. ex Kunth, *D. semperflorens* Uline, *D. triphylla* G.W.Schimp. ex A.Rich., and *D. paleata* Burkill. Morphological features of inventoried yam species are provided at Fig. 2. Taxonomic diversity analysis in Kabare and Kalehe revealed moderate diversity based on the Shannon-Wiener index (H’=1.78–2.02); with a Dominance index (D) varying between 0.15 and 0.25 and a Pielou equitability index (J) of 0.74 to 0.88, indicating well-balanced species distribution. No significant differences existed between Kabare and Kalehe (*p* = 0.096), implying that species were evenly distributed across both sites. At the provincial scale, species richness was highest in forest zones compared to savanna ecosystems (Fig. 3). This diversity in consumed yam species is likely linked to the region’s ethnic heterogeneity and diverse biophysical landscapes^19^, to the extent that, some species are exhibiting narrow distribution and are being consumed exclusively by specific communities. For instance, *D. dumetorum* is mostly cultivated by the Bashi, Bahavu, and Batembo ethnic groups in highlands and forest areas, while *D. alata* is predominant among the Babembe and Bafuliru ethnic groups in the lowland savannas of Uvira and Fizi (near the Lake Tanganyika). Meanwhile, wild and semi-domesticated yams such as *D. bulbifera* and *D. praehensilis* are predominantly consumed by Batwa pygmies living close by the Kahuzi Biega National Park^21^.

Historical account suggests that *D. alata* was introduced to Africa via Madagascar and Zanzibar coast by invading or colonizing Asiatics^16^, which could explain its presence in regions adjacent to the Lake Tanganyika, a historical trade and migration route in the DRC. Notably, an unexpected discovery of a yellow flesh *D. alata* landrace, locally called “Bigedja” on Idjwi Island in the Lake Kivu, raises questions about alternative introduction pathways. This variation in yam diversity across ethnic groups and agroecological zones underscores the importance of tailoring promotion efforts to local needs and preferences and environmental conditions. The only species widely known and consumed by all ethnic groups is *D. dumetorum* though ethnic groups seemed attached to particular landraces, making that *D. dumetorum* landraces are often designated by the origin or the ethnic group that maintain them^19^. Given this diversity, molecular fingerprinting is essential to ascertain species identity and refine genetic relationships among species and landraces grown by ethnic groups across the South-Kivu province.

### Socio-demographic information

The socio-economic characteristics of the surveyed population are detailed in Table **S2**. The surveyed population was dominated by men (60%) and middle-aged adults (40%), with farming as primary economic activity (95%), although some engaged in non-farm income-generating activities such as handicrafts and petty trading. The surveyed population had low levels of formal education, with over 85% having never attended school and 12% reaching only primary education level. Household sizes were typically large with 5–10 family members and nearly all participants lived far below the international poverty line, earning less than 1$ income a day. Most community members were affiliated with local community associations.

Yam consumptions in households were primarily derived from farming (62%), with smaller contributions from local markets (13%), forests (7.5%), or combination of both forests and farming (15%). When cultivated, yam is predominantly intercropped with other staple crops such as cassava, beans, taro, sweet potatoes, and maize, highlighting the yam integration into traditional agroecosytems^19^. Yam is mainly consumed boiled, the cooking time varying with species. It is noteworthy that though local communities are less aware of anti-nutritional factors in yam, cooking time varies with species. For instance, it takes hours to cook *D. dumetorum* while local *D. alata* varieties are safe to eat in less than an hour since the population perceives them less poisonous.

### Local knowledge on nutritional and health benefits

Local knowledge on nutritional and health benefits of yam was mainly assessed among autochthonous Pygmies and other ethnic groups residing near Kahuzi Biega National Park in Kabare and Kalehe territories. Despite the cultivation and consumption for food, only one-third (33%) of respondents were aware of the therapeutic benefits of yam, most associating it primarily with type 2 diabetes mellitus and stomach ache treatment. Local communities use yams to treat more than 15 health conditions, including stomach pains and food intoxications, skin wounds and infections, glycemic index, reproductive abnormalities, immunity system deficiency, respiratory challenges, diabetics, etc. though the effectiveness vary with used yam species and treatment procedures (Table 3). Yam plant parts involved in traditional medicine are boiled tubers, boiled leaves, and crushed leaf juice.

A recent study that listed yam among non-timber forest products in South-Kivu highlands reported that a third of the population around the Kahuzi Biega National Park use yam to improve digestion, to prevent prostatitis or as an anti-malaria treatment, while more than half knew yam as a type 2 diabetes’ prevention measure^21^. These anti-diabetic, anti-inflammatory, antimicrobial potentials, and their involvement in treating dozens of health conditions were also reported by several scholars^8–10,12–14,40^. It is noteworthy that traditional medicine is often the only healthcare available in some remote areas of South-Kivu where resource-poor households cannot afford modern medical expenses^21^. Of the ten listed yam species, *D. dumetorum* and *D. bulbifera* were more popular in traditional pharmacopeia in which different plant parts are used such as leaves, tubers, or bulbils either fresh, boiled, crushed, peeled, or incinerated. Even within a species, specific cultivars were recommended for particular health conditions (Table 3). This was particularly the case for *D. dumetorum* for which the yam origin was the main selection criterion, the origin being tightly associated with the genetic background of the cultivar being maintained by a particular ethnic group. Such effect of the genotype on *D. dumetorum* antidiabetic and antioxidant profiles was also reported by Aiyedun et al.^41^ in Nigeria. Laboratory experiments are necessary to confirm these perceptions by South-Kivu communities to determine bioactive compounds holding curative power for each species, to improve success rate of traditional medicine, to mitigate safety issues.

**Table 3:** Some diseases treated by yams and how they are treated.

Treated diseases	Treatment procedure
Malaria	Boil *D. dumetorum* yam leaves and take their juice twice a day
Abortion and sciatic nerves	Crush *D. bulbifera* bulbils to obtain a paw mass, mix it with water and boil, take one glass in the morning and one in the evening for nerves. To prevent abortion, take 1/2 glass a day.
Intestinal worms and stomach aches	Boil *D. dumetorum* yams for 24 h, adding water three times, then eat. Alternatively, crush raw *D. dumetorum* tubers or crush *D. dumetorum* leaves and drink the juice. Eat yams regularly
Hemorrhaging	Put a drop of juice from crushed *D. dumetorum* yam leaves into the bleeding nose.
Physical weakness (asthenia)	Eat boiled yams regularly whether it is wild or *D. dumetorum* yams
Hyperglycemia (diabetes mellitus)	Boil tubers, especially *D. dumetorum* yams, and eat them regularly. Dry and pound *D. dumetorum* to obtain a flour, eaten daily as fufu.
“Ngongo” finger disease	Use the *D. bulbifera* bulbil after crushing it and then putting the paw on the diseased part of the finger.
Rheumatism	Boil *D. dumetorum* leaves and use its juice as tea
Otitis	Part of *D. dumetorum* leaves are crushed and another part is boiled in water, then a drop of juice from the boiled leaves and a drop of crushed leaves’ juice are put in the infected ear.
Amoeba	*Dioscorea praehensilis* leaves are prepared as a vegetable (boiled in water) and then eaten as a snack.
Liver cirrhosis	Boil red *D. bulbifera* bulbil in water until ready, and then take its red juice as a tea twice a day.
Immune system deficiency	Eat yams as a meal on a regular basis
Diarrhea	Mash *D. dumetorum* leaves with those of *Psidium guajava* L. and *Galinsoga quadriradiata* Ruiz & Pav. and drink the juice.
Furuncle (skin abscess)	Use the *D. bulbifera* bulbils, peel and place the inner part of the bulbils on the boil (the white part). Crush *D. bulbifera* leaves or bulbils and apply to abscess.
Hidden hunger (anemia)	Eat yams regularly as a meal
Vomiting	Mash *D. dumetorum* leaves and drink their juice
Burns with fire	Incinerate *D. bulbifera* bulbils and apply to wounds.
Bronchitis and pneumonia	Crush the tuber and make it a drink.

Yam is also used in traditional rituals; for the Batwa pygmies, yams are seen as a means of communicating with their ancestors. They prepare wild yams and eat them in their traditional caves. For the Bashi tribe, boiled yams are used as a means of defense and to have the power to win a trial. Therefore, in some conservative zones, people carrying yams are not allowed to visit prisoners worrying that it will confer them supernatural power for release. For the Tembo tribe, the dry yam vine is used in the enthronement of the king to symbolize strength and power. Culturally yams are used to welcome high profile visitors across ethnic groups. Some wild yams such as *D. praehensilis* are believed to protect against witchcraft. Use of yam for rituals have been widely reported by the literature^2^.

### Physicochemical compositions of major yam species

#### Proximate composition and energy content

The physicochemical composition of yams varied significantly across species (*p* < 0.01, Table 4), with distinct differences observed in moisture, ash, protein, fiber, fat, carbohydrate, and energy. *Dioscorea dumetorum* exhibited the highest protein content (8.7 g/100 g), followed by purple *D. alata* (6.2 g/100 g) whereas white *D. alata* had the lowest protein content (4.3 g/100 g). Ash content was highest in white *D. alata* (2.9 g/100 g), followed by *D. bulbifera*’s underground (2.6 g/100 g) and aerial bulbils (2.3 g/100 g), while *D. dumetorum* had the lowest (2.0 g/100 g). Fiber content was the highest in *D. dumetorum* (4.2 g/100 g), followed by *D. bulbifera* aerial bulbils (3.4 g/100 g), whereas *D. praehensilis* had the lowest (1.2 g/100 g). Fat content was the highest in *D. praehensilis* (1.9 g/100 g) and purple *D. alata* (1.8 g/100 g). In terms of carbohydrate, *D. praehensilis* had the highest content (80.9 g/100 g), followed by white *D. alata* (79.4 g/100 g) and *D. bulbifera* underground tubers (78. 9 g/100 g). Energy content was also the highest in *D. praehensilis* (358.23 Kcal/100 g) and white *D. alata* (349.06 Kcal/100 g).

This richness of yams in proteins, fiber, and energy qualify them as nutritious healthy foods. For instance, yam contents in proteins and carbohydrates are superior to values reported by Irakiza et al.^42^ for cassava, the major tuber crop in eastern DRC. High fiber content highlights yam importance for digestive health, as fiber improves intestinal function and helps regulate intestinal transit. Yam low fat could explain its potential to regulate glycemic index and blood sugar among insulin-dependent patients^40^.

**Table 4:** Proximate composition and energy content of yam species.

Species	Moisture (g/100 g)	Ash (g/100 g)	Protein (g/100 g)	Fiber (g/100 g)	Fat (g/100 g)	Carbohydrate (g/100 g)	Energy (Kcal/100 g)
*D. bulbifera* (U)	10.2 ± 0.4ab	2.6 ± 0.2b	4.8 ± 0.3 cd	2.6 ± 0.1c	0.9 ± 0.1e	78.9 ± 0.2b	342.8 ± 2.7 cd
*D. alata* (W)	10.1 ± 0.9ab	2.9 ± 0.0a	4.3 ± 0.2d	1.7 ± 0.1d	1.6 ± 0.1b	79.4 ± 0.8b	349.1 ± 3.4b
*D. alata* (P)	10.2 ± 0.2ab	2.1 ± 0.1 cd	6.2 ± 0.4b	3.2 ± 0.2b	1.8 ± 0.0a	76.5 ± 0.4c	346.7 ± 1.9bc
*D. bulbifera* (A)	10.8 ± 0.3a	2.3 ± 0.1c	5.1 ± 0.2c	3.4 ± 0.1b	1.2 ± 0.0c	77.2 ± 0.2c	339.9 ± 1.0d
*D. dumetorum*	9.0 ± 0.4c	2.0 ± 0.2d	8.7 ± 0.3a	4.2 ± 0.4a	1.0 ± 0.1d	75.2 ± 0.9d	344.4 ± 2.9c
*D. praehensilis*	9.4 ± 0.5bc	2.1 ± 0.1 cd	4.7 ± 0.3 cd	1.2 ± 0.0e	1.8 ± 0.1a	80.9 ± 0.9a	358.2 ± 2.1a
*P-value*	0.009**	< 0.001***	< 0.001***	< 0.001***	< 0.001***	< 0.001***	< 0.001***

Results are presented as mean ± standard deviation (*n* = 3). Values with different letters in the same column are significantly different at p-value threshold of 0.05 of the LSD test. **, ***: highly significant and very highly significant at *p* < 0.01 and *p* < 0.001, respectively. U = underground tuber, W = white tuber flesh color, P = purple tuber flesh color, A = aerial tuber.

#### Mineral composition of yams

Except for the zinc, other mineral and β-carotene contents differed significantly among yam species (*p* < 0.01, Table 5). *Dioscorea dumetorum* exhibited the highest potassium content (240.2 mg/100 g), whereas *D. bulbifera* underground tubers had the lowest (107.4 mg/100 g). The highest magnesium content was recorded on *D. praehensilis* (125.9 mg/100 g) and *D. dumetorum* (121.84 mg/100 g) while white *D. alata* had the lowest amount (71.0 mg/100 g). A similar trend was observed for iron concentration; with *D. praehensilis* having the highest level (5.2 mg/100 g), followed by *D. bulbifera* aerial bulbils (5.2 mg/100 g), whereas white *D. alata* had the lowest (3.9 mg/100 g). The calcium content was highest in *D. bulbifera* underground tubers (40.1 mg/100 g), followed by the white *D. alata* (31.8 mg/100 g), while purple *D. alata* had the lowest calcium content (11.2 mg/100 g). In terms of β-carotene, *D. bulbifera* underground tubers (1.9 mg/100 g) and purple *D. alata* (1.8 mg/100 g) contained the highest levels, whereas *D. praehensilis* had the least amount (0.5 mg/100 g). High contents in iron, zinc, and β-carotene as compared to cassava showed yam potential to alleviate hidden hunger that constitutes a major public concern, especially among women and children, in eastern DRC^21,42^. Besides, its high content in potassium contributes to its potential in regulating glycemic index and blood sugar among insulin-dependent patients^40^. Differences in mineral compositions among species show that each yam species has a unique mineral profile, which may influence their nutritional and therapeutic use. Yam species richness in potassium and magnesium, such as *D. dumetorum* and *D. praehensilis*, may be particularly beneficial for cardiovascular health. Similarly, species with high iron contents, such as *D. praehensilis* could prevent anemia^43^.

**Table 5:** Mineral composition of wild and cultivated Yam species.

Species	Zinc (mg/100 g)	Potassium (mg/100 g)	Magnesium (mg/100 g)	Iron (mg/100 g)	Calcium (mg/100 g)	β-carotene (mg/100 g)
*D. bulbifera* (U)	2.2 ± 0.2	107.4 ± 8.8d	91.4 ± 5.1c	4.7 ± 0.2a	40.1 ± 2.2a	1.9 ± 0.0a
*D. alata* (W)	2.2 ± 0.2	107.5 ± 3.4d	71.0 ± 2.4d	3.9 ± 0.3b	31.8 ± 3.0b	0.5 ± 0.0e
*D. alata* (P)	2.1 ± 0.2	132.3 ± 10.8 cd	107.3 ± 5.4b	5.1 ± 0.3a	11.2 ± 0.9e	1.8 ± 0.0b
*D. bulbifera* (A)	2.2 ± 0.3	183.6 ± 26.2b	117.6 ± 8.8ab	5.2 ± 0.3a	15.6 ± 0.6d	1.0 ± 0.0d
*D. dumetorum*	1.8 ± 0.1	240.2 ± 18.3a	121.8 ± 12.7ab	5.0 ± 0.5a	23.5 ± 1.3c	1.0 ± 0.0c
*D. praehensilis*	1.9 ± 0.0	143.2 ± 11.9c	125.9 ± 10.2a	5.2 ± 0.4a	23.8 ± 0.6c	0.5 ± 0.0f
*P-value*	*0.07ns*	< 0.001***	< 0.001***	0.004**	< 0.001***	< 0.001***

Results are presented as mean ± standard deviation (*n* = 3). Values with different letters in the same column are significantly different at p-value threshold of 0.05 of the LSD test. ns: not significant (*p* > 0.05), **, ***: highly significant and very highly significant at *p* < 0.01 and *p* < 0.001, respectively. U = underground tuber, W = white tuber flesh color, P = purple tuber flesh color, A = aerial tuber.

#### Qualitative phytochemical composition of yams

Qualitative phytonutrient composition analyses showed high levels of alkaloids, terpenoids, and saponins in all test yam species though each species had a unique profile of bioactive compounds, which may influence their potential use in nutrition and medicine. Saponins and terpenoids were in largest amounts in purple *D. alata* and *D. dumetorum* and lowest in *D. bulbifera* underground tubers. These yam species rich in saponins and terpenoids could be particularly beneficial for their antioxidant and antimicrobial properties^8,10,40^. Purple *D. alata* had as well the highest amounts of alkaloids while all other species had this phytochemical in moderate amounts. Richness in alkaloids could confer purple *D. alata* interesting pharmacological applications. It is noteworthy that the balance between these bioactive compounds is crucial to maximizing health benefits, implying that diversified consumption of different yam species could offer a full range of nutrients and bioactive compounds, contributing to better overall health^44^. Other bioactive compounds with health benefits found in yams include Diosgenin, Dioscin, Protodioscin, Gracillin, Protogracillin, etc^14^., though they were not assessed in this study.

#### Phytonutrient composition of yams

Quantitative phytochemical compositions varied significantly with yam species (*p* < 0.001, Table 6), prompting an influence on their nutritional and health benefits. Flavonoid contents were highest in purple *D. alata* (0.96 mg/100 g), followed by *D. dumetorum* (0.85 mg/100 g) while *D. bulbifera* underground tubers had the lowest concentration (0.23 mg/100 g). The trend was similar for phenols as purple *D. alata* (0.99 mg/100 g) and *D. dumetorum* (0.61 mg/100 g) outperformed other species, with *D. bulbifera* aerial and underground tubers recording the lowest amounts (0.33 mg/100 g). It is noteworthy that species rich in flavonoids and phenolic compounds, such as purple *D. alata*, could offer better protection against cardiovascular disease and oxidative stress. However, species with high tannin content, such as *D. bulbifera* aerial bulbils (252.14 mg/100 g) and white *D. alata* (252.01 mg/100 g), may require specific preparation to minimize the potential tannins effects on essential minerals’ bioavailability^43^.

**Table 6:** Quantitative phytochemical composition of wild and cultivated yams.

Species	Flavonoids (mg/100 g)	Phenols (mg/100 g)	Tannins (mg/100 g)
*D. bulbifera* (U)	0.23 ± 0.01d	0.33 ± 0.00e	144.64 ± 2.87b
*D. alata* (W)	0.41 ± 0.02c	0.58 ± 0.01c	252.01 ± 3.00a
*D. alata* (P)	0.96 ± 0.05a	0.99 ± 0.01a	73.69 ± 0.50d
*D. bulbifera* (A)	0.42 ± 0.02c	0.33 ± 0.01e	252.14 ± 10.05a
*D. dumetorum*	0.85 ± 0.02b	0.61 ± 0.00b	49.74 ± 0.94e
*D. praehensilis*	0.38 ± 0.01c	0.49 ± 0.01d	91.61 ± 2.33c
*P-value*	< 0.001***	< 0.001***	< 0.001***

Results are presented as mean ± standard deviation (*n* = 3). Values with different letters in the same column are significantly different at 0.05 p-value threshold of the LSD test. ***: significant at *p* < 0.001. U = underground tuber, W = white tuber flesh color, P = purple tuber flesh color, A = aerial tuber.

#### Free radical scavenging activity

**Fig. 4:**
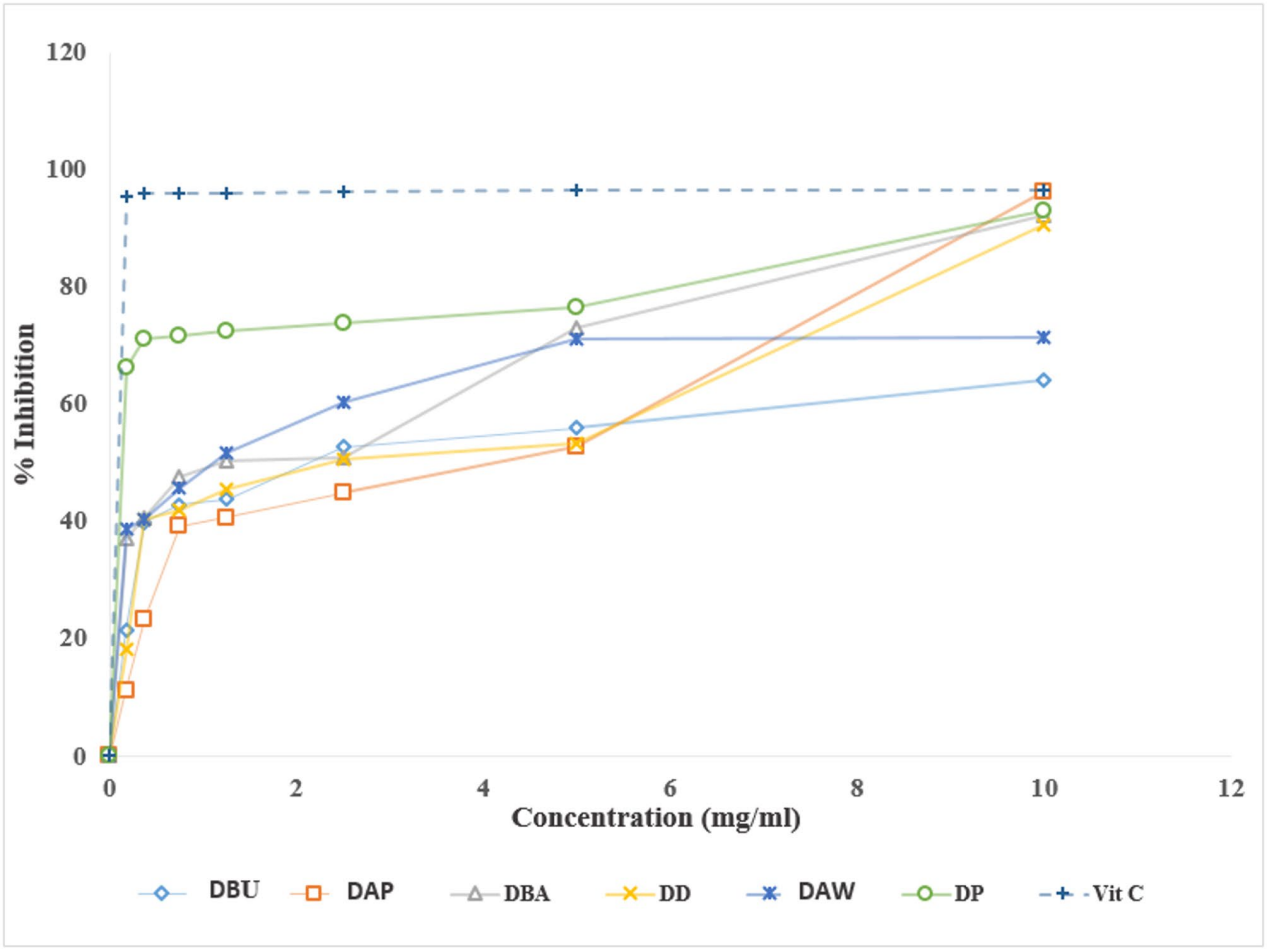
Free radical scavenging activity for yam species. DBU: *D. bulbifera* (Underground tuber); DAP: *D. alata* (Purple tuber flesh color); DBA: *D. bulbifera* (Aerial tuber); DD: *D. dumetorum*; DAW: *D. alata* (White tuber flesh color); DP: *D. praehensilis*, Vit C: Vitamin C.

Test yam species had varying antioxidant capabilities (Fig. 4). For instance, *D. praehensilis* and purple *D. alata* had very high antioxidant activities, almost reaching the level of vitamin C, with inhibition levels exceeding 90% at higher concentrations. Such species, with higher inhibition levels, could be valuable natural sources of antioxidants for food or medical applications^45^. *Dioscorea bulbifera* aerial bulbils and *D. dumetorum* had moderate antioxidant activity while white *D. alata* and *D. bulbifera* underground tubers showed lower inhibitions, indicating a lesser capacity to neutralize free radicals.

**Fig. 5:**
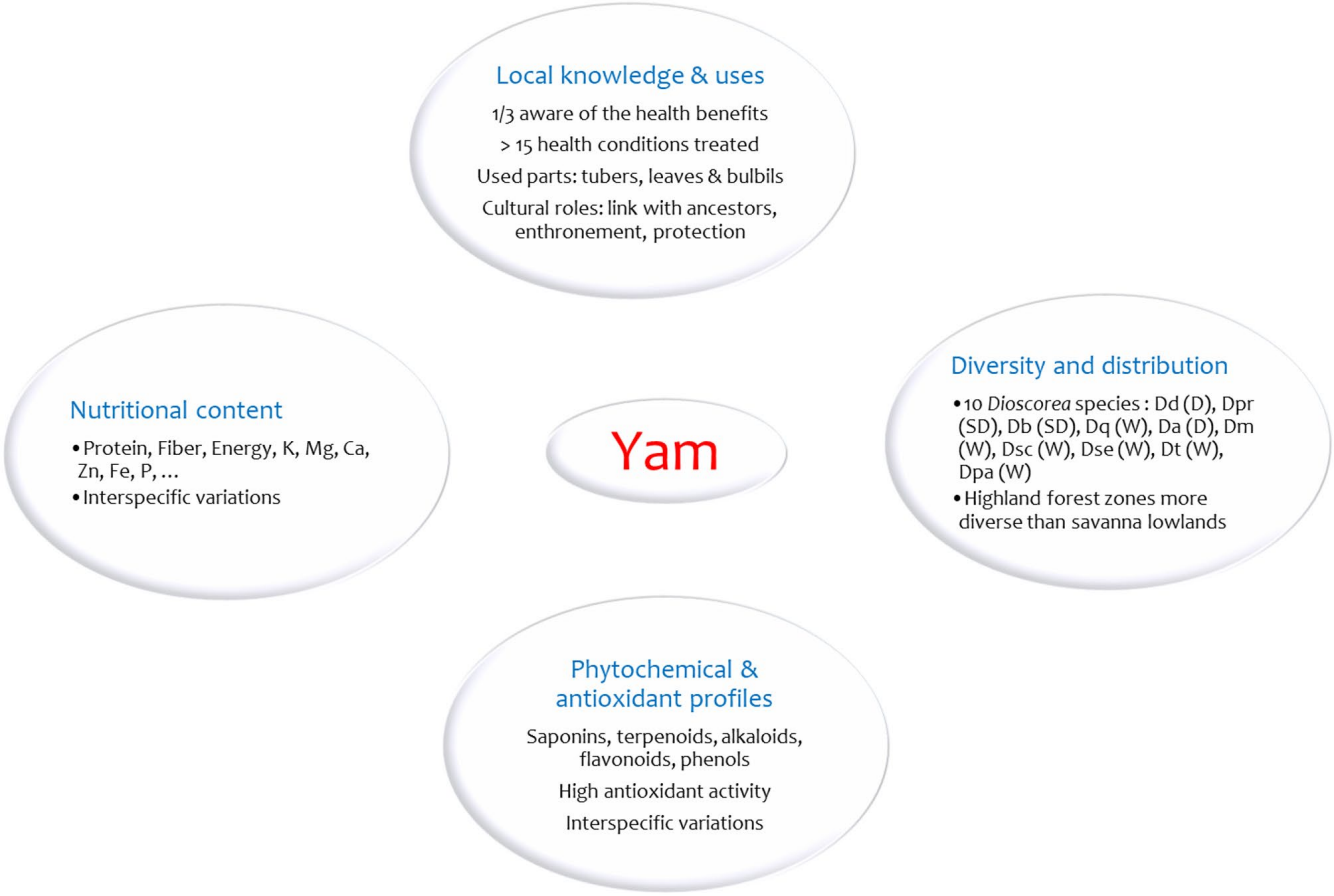
Summary results of yam diversity, nutritional and health benefits in eastern DRC. Dd: *D. dumetorum*, Dpr: *D. praehensilis*, Db: *D. bulbifera*, Dq: *D. quartiniana*, Da: *D. alata*, Dse: *D. semperflorens*, Dsc: *D. schimperiana*, Dm: *D. minutiflora*, Dpa: *D. paleata.* Letters in brackets refer to D: domesticated, SD: semi-domesticated and W: wild.

## Study limitations and prospects

Identification of species were based on morphological features that may lead to misclassification of species with limited distinctive morphological characteristics. Molecular fingerprinting is essential to ascertain species identity and refine genetic relationships among species and landraces grown by ethnic groups across the South-Kivu province. Future studies should complement perceptions of local communities on health benefits by laboratory experiments to determine bioactive compounds holding curative power for each species, to improve success rate of traditional medicine, and to mitigate safety issues. Special attention should be oriented towards the effectiveness of local yam species to treat chronic diseases such as diabetes and high blood pressure, a major public health concern in eastern DRC^46^. Investigating additional agroecological zones with different ethnic groups could enrich the list of consumed yam species and related nutritional, health, and cultural significance. Due to limited funds, the physicochemical composition analysis was only performed on the four most popular yam species.

## Conclusions

This study associated field observations, local knowledge, and laboratory essays to generate sound scientific knowledge on yam diversity, nutritional and health benefits of yam in eastern DRC. We documented 10 cultivated and semi-domesticated yam species consumed in eastern DRC, with distribution patterns shaped by biophysical conditions and cultural factors. These species are not only sources of food and income, but also play an important role in traditional medicine as they are involved in treating dozens of health conditions (Fig. 5). Physicochemical analyses showed that local yam species are valuable sources of nutrients and bioactive compounds that contribute to nutritious healthy diets in the region. However, nutritional and health benefits differ with yam species; implying that diversifying consumption with different yam species could offer a full range of nutrients and bioactive compounds to contribute to better overall health. While purple *D. alata* and *D. dumetorum* are particularly rich in bioactive compounds and secondary metabolites with limited anti-nutritional factors, *D. praehensilis* and purple *D. alata* demonstrated highest antioxidant activity. These potentials positioned these yam species as valuable inputs for pharmaceutical applications. Additional investigations are necessary to (1) extent physicochemical analyses to other identified yam species and varieties within species in the region; (2) extract bioactive compounds for pharmaceutical use; (3) assess local yam processing methods’ impacts on yam nutritional value, phytonutrient content, and antioxidant activity; and (4) develop domestication protocols for more promising wild and semi-domesticated yam species to ease pressure on natural forest reserves.

## Supplementary Information

Below is the link to the electronic supplementary material.

Supplementary Material 1

Supplementary Material 2

## Data Availability

The datasets used and/or analysed during the current study available from the corresponding author on reasonable request.

## Abbreviations

ANOVA: Analysis of Variance
AOAC: Association of Official Analytical Chemists
CRSN: Centre de Recherche en Sciences Naturelles
DRC: Democratic Republic of Congo
LSD: Least Significant Difference
UEA: Université Evangélique en Afrique
