# Supplementary material for: Nutritional and health benefits of wild and cultivated yam (Dioscorea spp.) species consumed in eastern Democratic Republic of Congo
Source: Sci Rep. 2025 Nov 26;15:42131. doi: 10.1038/s41598-025-26046-5 (PMC12658234; doi:10.1038/s41598-025-26046-5)
Supplement: Supplementary file 1 — Supplementary Material 1 [file 41598_2025_26046_MOESM1_ESM.docx]

**Proximate analysis**

Moisture, protein, fat, crude fiber and ash were determined according to (AOAC 2003) methods.

**Determination of moisture content**

The moisture dishes were washed and placed in a cabinet drier at 105°C for one hour. They were then the initial weight of the dish recorded afterwards (W1). Two grams of sample were taken and placed in the moisture dish and weight recorded (W2). The dishes were then placed in a cabinet drier for 3 hours. After the dying time the dishes were removed from the drier, cooled in a desiccator and the final weight recorded (. The moisture contents of the samples were calculated as shown below;

Moisture content (%) = (W3 – W1)/ (W2 – W1) × 10

**Determination of crude protein**.

One gram of the sample was weighed into a digestion flask together with a catalyst composed of 5 g of K2SO4 and 0.5 g of CuSO4 and 15ml of concentrated H2SO4. The mixture was heated in a fume hood till the digest color turned blue signifying the end of the digestion process. The digest was cooled, transferred into a 100 ml volumetric flask and topped up to the mark with distilled water. A blank digestion with the catalysts and acid was also made. Ten (10) ml of diluted digest was transferred into a distilling flask and washed with about 2 ml of distilled water. Distillation was done to a volume of about 60 ml distillate. The distillate was titrated using 0.02N-HCL to an orange color of the mixed indicator which signified the end point.

Calculations were done using the formulae below; Nitrogen%= (V1 –V2) × N × f × 0.014 × 100/V × 100/S Where; V1= Titre for the sample (ml); V2-titre for the blank N=Normality of standard HCL solution

F= Factor of standard HCL solution

V= Volume of diluted digest taken for distillation (10ml) S=Weight of sample taken (g)

Protein %= Nitrogen × protein factor

**Determination of crude fat**

The soxhlet extraction method which gives intermittent extraction of oil with excess of fresh organic solvent was used. Five grams of samples were weighed into extraction thimbles and the initial weights of the extraction flask taken. Fat extraction was done using petroleum ether in soxhlet extraction apparatus for 16 hours. The extraction solvents were evaporated and the extracted fat dried in an oven for about 15 min before the final weights of the flasks with the extracted fat were taken.

Calculations were done using the formula below;


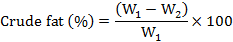


Where;

W1= Weight of sample before extraction

W2=Weight of sample after extraction

**Determination of crude fibre**

Two grams of sample was weighed into a 500 ml conical flask. 200 ml of boiling 1.25% H2SO4 was added and boiling done for 30 minutes under a reflux condenser. Filtration was done under slight vacuum with Pyrex glass filter and the residue washed to completely remove the acid with boiling water. 200 ml of boiling 1.25% NaOH was added to the washed residue and boiling done under reflux for another 30 minutes. Filtration was done using the same glass filter previously used with the acid. The residue was rinsed with boiling water followed by 1% HCL and again washed with boiling water to rinse acid from the residue. The residue was washed twice with ethanol and thrice with pet ether. It was them dried in an oven at 105°C in a porcelain dish with a constant weight (W1). Incineration was done in a muffle furnace at 550°C for 3 hours, the dish was then cooled in a desiccator and the final weight (W2) taken.

Calculations were done as shown below;


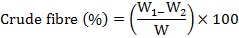


Where;

W1= Weight of acid and alkali digested sample

W2= Weight of incinerated sample after acid and alkali digestion

W= Weight of sample

**Determination of ash**

Samples weighing 2 g were first weighed and put into clean and dry crucibles The samples were the charred by flame to eliminate organic material before being incinerated at 550°C in a muffle furnace to the point of white ash. The residues were cooled in desiccators and weights taken.

Calculations were done as shown below:


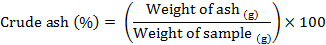


**Total available Carbohydrates**

The content of total carbohydrate was calculated by subtracting the sum of moisture, protein, fat, ash and crude fibre from 100% as shown below.

Total available carbohydrate = 100% - (moisture + protein + fat + ash + crude fibre)%

### Determination of Flavonoids

**Qualitative analysis**: this was done according to the method of Harborne (1973); 5 mL of dilute ammonia solution was added to a portion of aqueous filtrate of extract followed by addition of concentrated H_2_SO_4_. A yellow coloration observed indicated the presence of flavonoids. The yellow coloration disappeared on standing.

**Quantitative analysis**: Aluminum chloride colorimetric method was used for determination of flavonoids (Jagadish et al., 2009). To 10 mL volumetric flask 4 mL of distilled water and 1 mL of plant extract were added. After 3 minutes, 0.3 mL of 5 % sodium nitrite solution was added. After 3 minutes, 0.3 mL of 10 % aluminum chloride was added. After 5 minutes, 2 mL of 1 M sodium hydroxide was added and the volume made up to 10 mL with water. Absorbance was measured at 415 nm using UV-Vis spectrophotometer (Shimadzu model UV –VIS 1800, Kyoto, Japan). The amount of total flavonoids was calculated from Calibration curve of standard prepared from quercetin.

**Determination of total polyphenols**

Determination of total polyphenol was carried out using the method of waterman and mole with modification (1994) 1 gram of sample and put in amber bottle glass bottle, 50 ml of methanol was added and secure properly, then extracted for 3 hours in a shaker. The extract was kept in the dark for 72 hours for further extraction then filtered using Whatman number four, then topped up the extract to 50ml using methanol. centrifuge the extract for 10 min at 25 degrees at 150 rpm, I ml of the supernatant was taken and filter using 0.45 ul micro filter, put in a test tube then 2 ml of folin ciocateu 10% was added, Vortex and 4 ml of 0.7M sodium carbonate was added then Vortexed again, leave the extract for two hours to develop color, absorbance was read at 765 nm using gallic acid as a standard at Uv-Vis machine.

### Determination of Minerals

Analyses were done by dry ashing and atomic absorption spectrophotometer (AAS) (Shimadzu AA-7000), according to AOAC, (1984); Osborne and Voogt, (1978).

Clean dry crucible was weighed and about 5 grams of sample weighed into it. The crucibles were placed on a hot plate under a fume hood and the temperature increased slowly until smoking cease and the samples were thoroughly charred. They were then put in muffle furnace and temperature increased gradually to 250^0^ and heated for 1 hour. The temperature was increased to 600^0^ and incinerated for about 6 hours. The temperature was then decreased to 300^0,^ the crucibles removed and cooled to room temperature. The ash was transferred quantitatively to 100 ml beaker using 20 mL of 0.5N HNO_3_, then heated at 80-90^0^C on a hot plate for 5 minutes. This was then transferred to 100 mL volumetric flask and filled to the mark using 0.5N HNO_3_. Insoluble matter was filtered and the filtrate kept in a labeled polyethylene bottle. The absorbance of the solutions was read by Atomic Absorption Spectrophotometer (AAS). The various mineral standards were also prepared to make the calibration curve.

**DETERMINATION OF Β-CAROTENE CONTENT**

Weigh accurately approximately 2 g of fresh material. Chop finery and place in a motor with about 10 ml of acetone. Grind thoroughly and transfer the acetone extract to a 100 ml volumetric flask. Extract the residue again with 10 ml of acetone and transfer the extract to the volumetric flask. Continue the extraction with acetone until the residue no longer gives color to the acetone. Make the combined extract to 100 ml.

Evaporate 25 ml of the extract to dryness on a rotary evaporator. Dissolve the residue in about 1 ml of petroleum ether and introduce the solution into a chromatographic column.

Elute with petroleum spirit and collect the β-carotene in a flask. Beta carotene goes through the column as a yellow pigment very quickly.

Make the β-carotene elute to a volume in the 25 ml volumetric flask with petroleum ether and read absorbance at 450 nm. Read the concentration of β-carotene from the standard curve and calculate the β-carotene content of the vegetable per 100g of material.

Where A= absorbance; volume = total volume of extract (25 ml); A1%1cm = absorption coefficient of total carotenoid in PE (2592).

### 3.7.3 Determination of the free radical scavenging activity

The radical scavenging activities of the plant extracts against 2, 2-Diphenyl-1-picryl hydrazyl (DPPH) radical (Sigma-Aldrich) were determined by UV spectrophotometer at 517 nm (Molyneux, 2003). The following concentrations of the extracts were prepared, 0.01, 0.1, 1.0, 2.0 and 5 mg/mL in methanol (Analar grade). Vitamins C were used as the antioxidant standard at concentrations of same as the extract concentrations. 1 mL of the extract was placed in a test tube, and 3 mL of methanol was added followed by 0.5 mL of 1 mM DPPH in methanol. A blank solution was prepared containing the same amount of methanol and DPPH. Methanol was used to zero the spectrophotometer and the absorbances were read at 517 nm after 5 minutes in UV-Vis spectrophotometer (Shimadzu model UV – 1601 PC, Kyoto, Japan). The radical scavenging activity was calculated using the following formula:

% inhibition of DPPH = {(A_B_ – A_A_)/A_B_} x 100

Where A_B_ is the absorption of blank sample and A_A_ is the absorption of tested extract solution. The results were expressed as percentage inhibition of DPPH and mean inhibitory concentrations

**Phytochemicals Screening of the Plant Extracts Determination of Qualitative Phytochemicals**

**Test for Alkaloids**

Five grammes of each plant extract was mixed with 5 ml of 1% (v/v) aqueous hydrochloric acid on a steam bath, 1ml of the filtrate was treated with few drops of Draggendoff’s reagent. Blue-black turbidity serves as preliminary evidence of alkaloids presence.

**Test for the Presence of Terpenoids (Salkowski test)**

Five millilitre of each plant extract was mixed in 2 ml of chloroform 100% (v/v), and absolute H2SO4 (3 ml) was carefully added to form a layer. Formation of a reddish-brown layer at the interface showed the presence of terpenoidsIC_50_) determined from a plot of % inhibition of DPPH versus concentration of extract.

**Test for Saponins**

Five grammes of each plant extracts were shaken with distilled water (5 ml) in a test tube. Frothing which persisted on warming was taken as preliminary evidence of the presence of saponins.

**4.5.3 Determination of tannin content**

Condensed tannins were assayed according to vanillin-hydrochloric acid method (Burns, 1971; Price, Van & Butler*,* 1978). Quarter a gram of ground sample was extracted with 10 ml of 4% HCl in Methanol by shaking for 20 min using a shaker (Labortechnik KS 250b, Germany) and separation done using a refrigerated centrifuge (Kokusan, Type H-2000C, Japan) at 4,500 rpm for 10 min at 25°C. The supernatant was put into a 25 ml volumetric flask and extraction from the residue was repeated with 5 ml of 1 % HCl in methanol. The second supernatant was combined with the first one and diluted to 25 ml. Standards were prepare using Catechin hydrate at 0, 10, 20, 40, 60, 80 and 100 µg/ml. Duplicate aliquots of 1 ml of sample extracts were put into test tubes where one served as sample blank. The samples and standard solutions were reacted with 5 ml vanillin-HCl reagent (prepared by mixing just before use, equal volumes of 8% HCl in methanol and 1% Vanillin in methanol) and allowed to stand for 20 min. To the samples blanks were added 5 ml of 4% HCl in methanol. Absorbance for all prepared solutions was read at 500 nm and tannin content calculated as percent catechin equivalent (CE) using the standard calibration curve.
