# Supplementary material for: Nutritional and health benefits of wild and cultivated yam (Dioscorea spp.) species consumed in eastern Democratic Republic of Congo
Source: Sci Rep. 2025 Nov 26;15:42131. doi: 10.1038/s41598-025-26046-5 (PMC12658234; doi:10.1038/s41598-025-26046-5)
Supplement: Supplementary file 2 — Supplementary Material 2 [file 41598_2025_26046_MOESM2_ESM.docx]

**Table S1.** Physical and chemical proprieties of soil in of the UEA Experimental Site at Kashusha compared to yam requirements

| **Parameters** | **Value** | **Requirement** |
| --- | --- | --- |
| pH-H_2_O [1:25] | 5.61 | 5.5–6.8 |
| Total nitrogen (TNK) [%] | 0.26 | 0.2–0.5 |
| PO_4_^3-^ [mg kg^-1^] | 127.50 | 10–30 |
| K^+^ [cmol kg^-1^] | 0.41 | 0.2–0.5 |
| Ca^2+^ [cmol kg^-1^] | 15.46 | 3–5 |
| Mg^2+^ [cmol kg^-1^] | 3.88 | 1–2 |
| Na^+^ [cmol kg^-1^] | 0.23 | ≤0.5 |
| CEC [cmol kg^-1^] | 17.60 | 10-25 |
| Al^3+^ [cmol kg^-1^] | 0.00 | ≤0.5 |
| Cl^-^ [meq kg^-1^] | 4.00 | ≤5 |
| Fe^3+^ [mg kg^-1^] | 2461.61 | 4–10 |
| Organic carbon ‘SOC’ [%] | 2.42 | 1.5-3 |
| Organic matter ‘SOM’ [%] | 4.18 | 2.5-5 |
| Sand [%] | 50.00 | 50–70 |
| Clay [%] | 26.00 | 15–30 |
| Silt [%] | 24.00 | 10–25 |
| Bulk density [g cm^-3^] | 0.61 | 1.1–1.5 |

**Table S2.** Socio-economic profile of indigenous wild yam farmers in Kabare and Kalehe

| **Variables**/**Modalities** | **Bitale (%)** | **Buyungule (%)** | **Katana (%)** | **Pooled (%)** | **Khi²** | **P-value** |
| --- | --- | --- | --- | --- | --- | --- |
| **Gender of household head** | | | | | | |
| Women | 43.33 | 26.67 | 46.67 | 40.00 | 1.527 | 0.465ns |
| Men | 56.67 | 73.33 | 53.33 | 60.00 |  |  |
| **Age of household head (years)** | | | | | | |
| < 30 | 0.00 | 0.00 | 13.33 | 3.33 | 9.402 | 0.152ns |
| > 50 | 40.00 | 40.00 | 26.67 | 36.67 |  |  |
| 30 – 40 | 16.67 | 33.33 | 33.33 | 25.00 |  |  |
| 40 – 50 | 43.33 | 26.67 | 26.67 | 35.00 |  |  |
| **Main activity** | | | | | | |
| Agriculture | 100.00 | 93.33 | 86.67 | 95.00 | 9.192 | 0.056* |
| Others | 0.00 | 6.67 | 0.00 | 1.67 |  |  |
| Small trade | 0.00 | 0.00 | 13.33 | 3.33 |  |  |
| **Education level** | | | | | | |
| No formal education | 93.33 | 73.33 | 80.00 | 85.00 | 5.100 | 0.277ns |
| Primary | 3.33 | 20.00 | 20.00 | 11.67 |  |  |
| High school | 3.33 | 6.67 | 0.00 | 3.33 |  |  |
| **Family size (members)** | | | | | | |
| < 5 | 6.67 | 13.33 | 6.67 | 8.33 | 4.815 | 0.306ns |
| > 10 | 13.33 | 0.00 | 0.00 | 6.67 |  |  |
| 5 – 10 | 80.00 | 86.67 | 93.33 | 85.00 |  |  |
| **Daily household income (CDF)** | | | | | | |
| > 5000 | 3.33 | 0.00 | 6.67 | 3.33 | 3.037 | 0.551ns |
| Low (< 2500 FC) | 90.00 | 86.67 | 93.33 | 90.00 |  |  |
| Moderate (2500 - 5000) | 6.67 | 13.33 | 0.00 | 6.67 |  |  |
| **Farming experience (years)** | | | | | | |
| 10 – 20 | 50.00 | 40.00 | 40.00 | 45.00 | 11.212 | 0.024* |
| 20 – 30 | 50.00 | 46.67 | 26.67 | 43.33 |  |  |
| 5 – 10 | 0.00 | 13.33 | 33.33 | 11.67 |  |  |
| **Membership of agricultural associations / cooperatives** | | | | | | |
| No | 63.33 | 13.33 | 40.00 | 45.00 | 10.303 | 0.005** |
| Yes | 36.67 | 86.67 | 60.00 | 55.00 |  |  |

*ns: not significant; ***: p<0.001; **: p<0.01; *: p<0.05; CDF: Congolese franc. At the time of the survey, $1 was 2800FC*
